# The “gut-immune-bone axis”: quantitative MRI reveals the microbial-immune-bone metabolic interplay in osteoporosis

**DOI:** 10.3389/fimmu.2026.1909663

**Published:** 2026-10-26

**Authors:** Liang Wu, Hong Yan Yao, Zhen Jiang Fu, Rong Hao Luo, Sheng Zhou

**Affiliations:** 1 Gansu University of Traditional Chinese Medicine, Lanzhou, China; 2 Gansu Provincial Hospital, Lanzhou, China

**Keywords:** bone metabolism, gut microbiota, gut-immune-bone axis, osteoimmunology, osteoporosis, quantitative magnetic resonance imaging, short-chain fatty acids

## Abstract

Osteoporosis is a systemic skeletal disorder, whose pathogenesis is related to abnormalities in bone metabolism, as well as gut microbiota and immune system dysregulations. This structured narrative review synthesizes recent evidence on the “gut-immune-bone axis” in osteoporosis, with emphasis on studies published from 2020 to 2025 and selected later studies incorporated during manuscript revision when directly relevant. This article first outlines the direct and indirect regulatory roles of gut microbiota and its products (short-chain fatty acids, tryptophan catabolites, etc.) in bone metabolism. It then focuses on immune cells that participate in bone remodeling, including the Th17/Treg balance, B cells, and macrophages, and their interactions with the gut microbiota. The review further discusses recent developments in quantitative magnetic resonance imaging for quantifying bone microarchitecture, marrow composition, and metabolic status and considers how these measures may serve as downstream phenotyping tools in studies of the gut–immune–bone axis. Finally, an integrative model of the microbial-immune-bone metabolic network is proposed and translational strategies, including microbiome-directed interventions, immunomodulatory therapies, and multi-omics approaches, are critically discussed.

## Introduction

Osteoporosis is a systemic skeletal disease in which the bone mineral density is reduced and the bone microarchitecture is degraded leading to an increase in bone fragility and fracture risk (1). There is a significant burden on individual health and socio-economic systems associated with the increasing prevalence and incidence of osteoporosis, due to the fact that the world is ageing (2). While a bone mineral density (BMD) T-score ≤ -2.5, measured by dual-energy X-ray absorptiometry (DXA), is still the diagnostic criterion, BMD alone is not completely representative of changes in bone microarchitecture and bone quality (3). In the last years, the focus has shifted from bone mass to other parameters including bone metabolic state, trabecular microarchitecture, and molecular biological modifications in bone as important determinants of bone fragility (4). In particular, a new idea, the “gut-immune-bone axis”, has been developed, which is a new regulatory system in the pathogenesis of osteoporosis. The intestinal microbiome is now regarded as a “hidden organ” that is communicating with host systems in a multitude of ways, including metabolites, immune signals and neuroendocrine pathways (5). Different studies have shown that changes in gut bacteria composition have a significant effect on bone metabolism and bone mass (6). At the same time, bone remodeling is tightly regulated by the immune system, creating a link between bone remodeling and the immune system which is bidirectional and defines the field of osteoimmunology (7). Thus, understanding the tripartite interaction between the gut, immune system and bone is essential to gain a global perspective of osteoporotic pathogenesis and to find new preventative and therapeutic approaches (8).

This review focuses on: (1) the effects of gut microbial composition and metabolites, such as short-chain fatty acids (SCFAs) and tryptophan catabolites (TCs), on bone cell function and metabolism; (2) the immunomodulatory function of the gut-bone axis, including T cells, B cells, macrophages, and Th17 cells and their role in osteoporosis; (3) quantitative magnetic resonance imaging (qMRI) techniques for osteoporosis evaluation and their potential to capture downstream changes in bone microarchitecture, marrow composition, and tissue characteristics; (4) an integrative model of the microbial-immune-bone metabolic network and research frontiers; and (5) the translation of preclinical findings, including microbial interventions, immune-targeted therapies, and imaging biomarkers. This article is a structured narrative review rather than a systematic review. We searched PubMed, Scopus, and Web of Science for literature published from January 2020 through December 2025 using combinations of terms including “osteoporosis”, “gut microbiota” or “microbiome”, “immune” or “osteoimmunology”, “microbial metabolites”, and “magnetic resonance imaging” or “quantitative MRI”. Eligible sources were peer-reviewed English-language original studies and high-quality reviews directly relevant to gut–immune–bone interactions, osteoporosis, microbial metabolites, osteoimmunology, or qMRI. Duplicates, non-peer-reviewed reports, and studies without direct skeletal relevance were excluded. Seminal earlier studies and selected studies published after 2025 were incorporated when necessary to establish mechanistic context or to update the revision. Study relevance, methodological quality, level of human evidence, and mechanistic contribution were prioritized; journal quartile and citation influence were considered only as secondary contextual indicators rather than formal eligibility criteria.

## The relationship between gut microbiota and bone metabolism

The gut microbiome plays a key role in maintaining bone metabolic homeostasis. Under physiological conditions, the commensal microbiota promotes the welfare of the skeleton in several ways; it helps the absorption of nutrients, maintains the integrity of the intestinal barrier and modifies the hormonal balance (9). For instance, a healthy microbiome has been shown to improve the absorption of minerals such as calcium and phosphorus, and produce healthy metabolites like B vitamins and vitamin K2 which can lead to better BMD (10). The gut microbiota also affects the levels of hormones that play an important role in bone metabolism such as estrogen, testosterone, insulin-like growth factor 1 (IGF-1), parathyroid hormone (PTH) and serotonin (11). For example, estrogen is key to maintenance of bone mass, which inhibits osteoclastogenesis and regulates osteoblast and osteoclast formation systemically and in the bone marrow niche by regulating T-cell homeostasis (12). Therefore, gut microbiota dysbiosis can negatively affect bone metabolism via multiple mechanisms (13).

Preclinical antibiotic-treated and germ-free models provide evidence supporting a causal contribution of the gut microbiota to skeletal phenotypes (14). In OVX mice, broad-spectrum antibiotic depletion of the gut microbiota reduced bone loss and preserved trabecular microarchitecture and bone strength (15), while transplantation of microbiota from osteoporotic OVX donors to recipient mice was sufficient to transfer a bone-loss phenotype (16). Another animal model showed that antibiotic-induced dysbiosis decreased BMD and that a fermented probiotic-containing milk product partly restored BMD and facilitated fracture healing (17). By contrast, current human evidence is predominantly observational: microbial diversity and the relative abundance of specific taxa have been associated with BMD and bone microstructural indices (18, 19), but these associations do not by themselves establish causality. Thus, animal, *in vitro*, and human findings are distinguished throughout this review, and mechanistic conclusions derived from germ-free, antibiotic-treated, OVX, or FMT models should not be directly extrapolated to human osteoporosis without prospective validation. Overall, available evidence suggests that the gut microbiota may influence bone remodeling through nutritional metabolism, barrier function, and immune homeostasis (20, 21) (**Table 1**).

**Table 1:** Representative human studies of the gut–immune–bone axis in osteoporosis.

Study/design	Population	Intervention/exposure	Microbiome findings	Immune/metabolic findings	Bone outcomes	Key conclusion
Wang et al., 2023 (22) Cross-sectional	98 postmenopausal women (PMO vs non-PMO)	No intervention	Altered bacterial/fungal diversity and differential fecal metabolites	Immune endpoints not assessed	Microbial/metabolite features correlated with BMD	Supports association, not causality, between gut signatures and PMO
Ma et al., 2024 (23) Cross-sectional	Postmenopausal women with vs without osteoporosis	No intervention	Reduced richness; altered taxa including lower butyrate-associated genera	↓ Tregs/IL-10; ↑ Th17, inflammatory cytokines and circulating LPS	Osteoporosis phenotype defined by BMD	Links dysbiosis, barrier dysfunction and immune imbalance in humans
Han et al., 2022 (24) Randomized trial	53 postmenopausal women	L. fermentum SRK414 vs control, 6 months	Changes in L. fermentum abundance were evaluated	Osteocalcin maintained; immune endpoints not assessed	Femoral-neck BMD increased in intervention group	Suggests possible strain-specific benefit; small trial requires replication
Gregori et al., 2024 (25) Randomized trial	239 early postmenopausal women	L. reuteri 6475 vs placebo, 2 years	Microbiome endpoints not central to trial	No significant effect on bone-turnover markers	No reduction in tibial, hip or spine bone loss	Demonstrates that probiotic efficacy is not consistent across trials
Yumol et al., 2025 (26) Randomized trial	72 postmenopausal women	L. brevis HA-112 + L. paracasei HA-274 vs placebo, 48 weeks	Microbiome endpoints not reported as primary outcomes	No sustained group benefit in measured bone-turnover markers	No significant improvement in femoral-neck BMD or FRAX risk	Supports cautious interpretation of probiotics as investigational adjuncts

PMO, postmenopausal osteoporosis; BMD, bone mineral density; LPS, lipopolysaccharide; FRAX, Fracture Risk Assessment Tool. “Not assessed” indicates that the endpoint was not directly measured rather than inferred mechanistically.

### Impact of gut microbial metabolites on bone cells

Much of the biological activity of the gut microbiota is through its metabolic products. Two particularly important groups of microbial metabolites with direct links to bone metabolism are short-chain fatty acids (SCFAs) and tryptophan catabolites (27).

1. Short Chain Fatty Acids (SCFAs). These SCFAs are formed by the process of anaerobic bacterial fermentation of dietary fiber in the colon and are mainly acetate (C2), propionate (C3) and butyrate (C4) (28). The SCFAs can be a very important source of energy to the colonocytes, and have various biological activities such as strengthening the intestinal mucosal barrier, and immunoregulation (29). Recent studies have shown that SCFAs have a significant positive impact on bone metabolism (30). Preclinical models of inflammation-associated and postmenopausal osteoporosis revealed that propionate and butyrate supplementation can significantly increase bone mass and prevent bone loss largely due to their ability to suppress osteoclastogenesis and bone resorption, without negatively affecting bone formation (31). Mechanistically, SCFAs cause metabolic change in osteoclast progenitors, increasing glycolysis and decreasing oxidative phosphorylation (32). This metabolic switch decreases the expression of important osteoclastogenic transcription factors such as TRAF6 and NFATc1 which directly impairs osteoclast differentiation and function and leads to a decrease in bone resorption (33). In addition, SCFAs such as butyrate are able to bind and/or activate G protein-coupled receptors (GPRs, e.g., GPR43, GPR41), which results in multiple signaling pathways being influenced and modulation of hormone secretion, inflammatory response and immune cell function (34). For example, SCFAs trigger glucagon-like peptide-1 (GLP-1) release from enteroendocrine L-cells and have indirect effects on skeletal glucose metabolism, and increase IGF-1 in the bloodstream, which increases bone formation (35). Specifically, SCFAs have been shown to have potent immunomodulatory properties, particularly able to induce the proliferation of regulatory T cells (Tregs) *in vivo* (36). Tregs are known to be anti-resorptive immune cells that exert inhibitory effect on osteoclastogenesis and augment bone mass accrual (37). Butyrate, by promoting the differentiation of colonic Tregs and subsequently increasing their prevalence in the bone marrow, is thought to exert a key bone-protective effect (38). Additionally, *in vitro* studies show that SCFAs can directly suppress inflammatory pathways; for example, sodium butyrate treatment inhibits NF-κB and ERK signaling in RAW264.7 macrophages, reducing pro-inflammatory cytokine levels (39), which also contributes to decreased osteoclast activity (40). Acid synthesis from short-chain fatty acids lowers intraluminal intestinal pH. Increased mineral solubility consequently facilitates calcium absorption by raising its bioavailability, which in turn promotes bone mineralisation indirectly (41). In summary, an adequate supply of SCFAs can promote bone formation and inhibit bone resorption through multiple pathways, thereby improving bone density and overall quality (42). Both animal studies and preliminary clinical trials support this premise: supplementation with exogenous SCFAs or promotion of endogenous SCFA production (e.g., via a high-fiber diet) improves bone turnover markers in postmenopausal women, positioning SCFAs as a promising novel interventional strategy for osteoporosis (43).

2. Tryptophan Metabolites. Tryptophan is a key substrate for microbial metabolism in the gut. Commensal bacteria convert tryptophan into indole and its derivatives (e.g., indole-3-acetic acid, indole propionic acid) and other metabolites, which can act as endogenous ligands for the aryl hydrocarbon receptor (AhR), thereby regulating intestinal immunity and mucosal barrier function (44). Under homeostatic conditions, continuous microbial production of tryptophan catabolites helps maintain intestinal immune homeostasis and epithelial integrity (45). However, in states of chronic inflammation or dysbiosis (e.g., inflammatory bowel disease, chronic colitis models), levels of tryptophan catabolites are markedly reduced, correlating with impaired intestinal barrier function, increased systemic inflammation, and often, significant bone loss (46). A study utilizing a mouse model of colitis-induced osteoporosis observed a significant reduction in microbial tryptophan metabolite levels in both serum and feces (47). This lack of tryptophan will lead to a breakdown in the barrier function of the intestinal epithelia, which will lead to a rise in its permeability to bacterial products and allow them to enter the systemic circulation (48). This, in turn, triggers a pro-inflammatory reaction in the bone marrow leading to bone loss (49). Fortunately, microbiota-induced recovery of tryptophan metabolism is a good remedy for this bone loss (50). Researchers have genetically modified a probiotic (Clostridium butyricum) bacterium to produce all of the metabolites of tryptophan in the gut continuously (51). This engineered probiotic was found to heal the intestinal mucosal barrier, decrease pro-inflammatory cytokines (TNF-α and IL-1β) and markedly improve bone mass (52). This synthetic biology approach prevented bone loss in mouse models of both chronic colitis and natural aging, suggesting that it could have therapeutic applications as a means to combat osteoporosis by targeting the intestinal tryptophan metabolic pathway (53). Microbial tryptophan catabolites act mainly through the AhR-mediated immune pathway, where activation of this receptor by a ligand leads to the activation of cells of the mucosal immune system (such as type 3 innate lymphoid cells, ILC3s) and secretion of cytokines, including IL-22, that promote production of antimicrobial peptides and mucins by the intestinal epithelial cells, thus enhancing the barrier and reducing inflammation (54). Better barrier function means fewer of these harmful molecules (such as lipopolysaccharide LPS) enters the system and fewer pro-inflammatory stimuli in the bone marrow microenvironment, which translates to less osteoclast activity (55). In addition, some metabolites of tryptophan might directly affect bone. For instance, indole propionic acid (IPA) has antioxidant and anti-inflammatory effects and is suggested to inhibit osteoclast formation and induce osteogenesis, but the exact mechanism has not been investigated (56). In summary, the tryptophan-microbiota metabolic axis is an important connection between intestinal inflammation and bone mass and its modulation by probiotics or by dietary interventions to increase the levels of tryptophan catabolites show promise as a complementary approach for osteoporosis prevention and intervention (57).

3. Other Microbial Metabolites. In addition to the production of SCFAs and tryptophan derivatives, the gut microbiota generates various other metabolites that may affect bone metabolism (58). Another notable group of molecules that are microbially modified are bile acids. Primary bile acids, synthesized in the liver and secreted into the intestine, are biotransformed by the gut microbiota into secondary bile acids (59). Research indicates that bile acids, beyond their role in lipid digestion and absorption, function as crucial signaling molecules capable of activating nuclear receptors like FXR and membrane-bound receptors such as TGR5 in distant tissues (60). These receptors are expressed in bone cells and mediate the skeletal effects of bile acids (61). For example, TGR5, a G protein-coupled bile acid receptor expressed on osteoblasts, can, upon activation, enhance the expression of Runx2, osteocalcin (OCN), and other osteogenic proteins (e.g., ALP, Osterix) via AMPK signaling, thereby promoting osteoblastic differentiation (62). Concurrently, FXR in bone positively regulates osteogenesis: FXR-deficient mice spontaneously develop a low bone mass phenotype (a 4–6% reduction in male mice between 8–20 weeks of age), whereas agonists like chenodeoxycholic acid (CDCA) and its derivatives can upregulate Runx2 through the ERK/β-catenin pathway, enhancing bone formation (63). Notably, co-activation of both FXR and TGR5 demonstrates a synergistic effect on osteogenesis compared to activation of either receptor alone (64). Moreover, specific microbially derived bile acids can modulate the osteoimmune environment (65). For instance, hyodeoxycholic acid (HDCA), a secondary bile acid produced by bacteria of the genus Parabacteroides, is found at diminished levels in aged mice and is associated with reduced bone mass (66). Supplementation with HDCA was shown to inhibit osteoclast maturation and activate TGR5 (67), thereby blocking NF-κB signaling and protecting bone microarchitecture *in vivo*; this protective effect was abrogated in TGR5-knockout mice (68). This discovery outlines an important ‘microbiota–HDCA–TGR5 axis’ that could be a target for treating age-related bone loss, and thus for osteoporosis (67). Moreover, vitamin K2 (menaquinones) is also being partially synthesized by intestinal bacteria. Various Bacteroides and Lactobacillus species can produce different menaquinone isoforms (69), and studies have shown that vitamin K_2_ supplementation reduces bone resorption markers and improves bone mass in OVX mice (70). Vitamin K promotes the differentiation of osteoblasts into osteocytes and inhibits osteoclastogenesis by activating bone matrix proteins such as osteocalcin and matrix Gla protein (71). Furthermore, gut-derived trimethylamine N-oxide (TMAO) is implicated in bone metabolism (72). Dietary choline is metabolized by gut microbes to trimethylamine (TMA), which is subsequently oxidized in the liver to TMAO (72). TMAO has been shown to inhibit the differentiation of bone marrow stromal cells into osteoclasts *in vitro*, an effect involving interference with the NF-κB pathway (73). Finally, the microbiota also generates polyamines (e.g., spermine, putrescine) and bacterial extracellular vesicles as novel factors capable of indirectly influencing bone remodeling by promoting intestinal epithelial proliferation and modulating immune responses (74). For example, outer membrane vesicles (10–400 nm in diameter) secreted by Escherichia coli can carry cargo such as miR-96-5p into host circulation, where they can inhibit osteoclast activity by affecting mitochondrial function and apoptotic pathways in osteoclasts, significantly reducing bone loss in OVX mice (75). These findings provide a new insight into how the gut microbiome can affect bone metabolism and point to the possibility of using probiotic-derived metabolites or engineered bacterial vesicles as new approaches to the treatment of osteoporosis (76).

Beyond these better-characterized pathways, an expanding microbial metabolome may influence bone through immune, metabolic, or direct skeletal mechanisms (77). Purine metabolites such as inosine, organic acids including succinate and lactate, phenolic microbial products, and microbial sphingolipids have been implicated in host immunometabolic regulation, but bone-specific causal evidence remains limited and is often derived from experimental systems. Of particular relevance to secondary osteoporosis, the gut-derived uremic toxins indoxyl sulfate and p-cresyl sulfate accumulate in chronic kidney disease and may contribute to CKD-related bone abnormalities by impairing osteoblast differentiation, altering Wnt-related signaling, and promoting low-turnover bone phenotypes (78). These emerging metabolites should therefore be interpreted according to disease context and level of evidence rather than assumed to have uniform effects across osteoporosis subtypes.

To sum up, the gut microbiota is linked to the skeleton through its metabolites and signaling molecules and its homeostasis is necessary for achieving normal bone remodeling (79). Under conditions of altered gut flora composition and/or metabolic activity, the equilibrium between bone formation and resorption may shift in favor of bone loss, leading to osteoporosis (80). A new generation of “gut microbiota-bone” axis intervention strategies is emerging, ranging from increasing dietary fiber to increase SCFA production, to using engineered probiotics to restore tryptophan metabolism, to targeting bile acid receptors with specific agonists (81), providing novel directions for osteoporosis prevention and treatment (82).

### Sex-specific immune responses and the gut–immune–bone axis

Sex represents an important biological variable in the gut–immune–bone axis and may contribute to substantial heterogeneity in osteoporosis pathogenesis. Although postmenopausal estrogen deficiency has been studied most extensively, osteoporosis in men is also influenced by aging, androgen deficiency, and alterations in sex-steroid signaling. Sex hormones regulate bone not only through direct effects on osteoblasts and osteoclasts but also by modifying intestinal microbial communities, barrier integrity, and systemic immune responses. Sex-dependent differences in innate and adaptive immunity may alter Th17/Treg balance, macrophage polarization, cytokine production, and the skeletal response to microbial metabolites. Experimental evidence further suggests that microbiome-mediated skeletal effects can be sex dependent. In young mice receiving gender-affirming hormone therapy, Pal et al. showed that hormone-shaped microbiota promoted intestinal Treg expansion, Treg migration to bone marrow, and bone formation in male but not female mice (83). This study provides mechanistic evidence for a sex-steroid–microbiome–immune interaction, but it is a preclinical model of skeletal maturation rather than direct evidence for male osteoporosis. Therefore, sex-stratified human cohorts and intervention trials are needed to determine whether gut microbiome signatures, immune phenotypes, and responses to microbiome-targeted therapies differ between women and men with osteoporosis.

### Clinical heterogeneity of osteoporosis and the gut–immune–bone axis

Osteoporosis is etiologically heterogeneous, and mechanisms established in estrogen-deficiency models should not be generalized to all clinical forms. Postmenopausal osteoporosis has the strongest mechanistic evidence for microbiome-dependent immune activation: in OVX mice, estrogen deficiency increases intestinal TNF-producing T cells and Th17 cells and promotes their trafficking to bone marrow, where TNF and IL-17 enhance osteoclastogenic signaling (84). In age-related osteoporosis, immunosenescence, barrier dysfunction, and age-associated changes in microbial metabolites may become more prominent, whereas glucocorticoid-induced osteoporosis additionally reflects direct suppression of osteoblastogenesis and altered marrow adiposity. Diabetic osteoporosis is shaped by metabolic inflammation and AGE/RAGE-related signaling, while CKD-associated bone disease is complicated by mineral metabolism, uremic toxins, and altered bone turnover. Chronic inflammatory disorders may involve persistent cytokine-driven osteoclast activation, and cancer therapy-associated bone loss may combine endocrine suppression, inflammation, and treatment-specific skeletal toxicity. Importantly, gut-to-bone immune trafficking is also context dependent. Butyrate-producing microbiota are required for the full anabolic skeletal response to intermittent PTH in mice (85), demonstrating that microbial effects may vary with endocrine signaling. Conversely, in a melanoma bone model, microbiome depletion accelerated intraosseous tumor growth and osteolysis by reducing intestinal NK- and Th1-cell trafficking to bone (86); this finding is relevant to tumor–bone immunology but should not be interpreted as direct evidence for primary osteoporosis. These examples underscore the need to stratify future studies by osteoporosis etiology, comorbidity, medication exposure, sex, age, and renal/metabolic status.

## The regulatory role of the immune system in gut-bone crosstalk

Beyond perturbations of bone metabolism, pathogenesis of osteoporosis involves also modifications of immune processes (87). The skeletal system and immune system appear to be closely tied in, with shared cytokines and signaling pathways, and seem to reciprocally regulate each other, a phenomenon referred to as osteoimmunology (88). The gut and the associated microbiota have a huge impact on the development of innate and adaptive immune system, which is closely related to bone remodeling immune regulation (89). The mechanistic role of T cells (specifically Th17 and Treg subsets), B cells and macrophages in the gut-bone axis will be outlined in this section (90). Importantly, the effect of the immune system on bone can be exacerbated by disruption of intestinal barrier. This includes the passage of bacterial products (91), including pathogen associated molecular patterns (PAMPs) like lipopolysaccharide (LPS), peptidoglycan (PGN), and flagellin, into the systemic circulation that is enhanced by increased intestinal permeability. These PAMPs act on pattern recognition receptors, such as TLRs and NOD-like receptors, on cells located in the mucosal epithelia as well as in bone marrow, leading to a systemic inflammatory response (92). This type of inflammatory signaling increases the activity of osteoclast precursors, which in turn leads to enhanced bone resorption, while at the same time inhibiting the activity of osteoblasts, which results in decreased bone formation (93), thus playing a crucial role in the pathogenesis of osteoporosis. There are known specialized roles for different types of immune cells and together they contribute to the net direction of bone remodeling through a complex cytokine network in the tripartite relationship between the gut, the immune system and the bones (87).

### T cells and their subsets: the Th17/Treg imbalance

T lymphocytes play a key role in osteoimmune regulation (94). CD4+ helper T cells (Th) can be polarized into different functional subsets such as Th1, Th2, Th17 and regulatory T cells (Tregs) that affect bone remodeling by production of particular cytokines (95). Of these, the balance of Th17 and Treg cells is of paramount importance with regard to bone homeostasis (96). Th17 cells are pro-inflammatory and pro-osteoclastogenic cells that secrete a potent pro-inflammatory cytokine called interleukin-17 (IL-17) (97). On the other hand, Treg cells release anti-inflammatory cytokines (such as IL-10 and TGF-β) and block the differentiation of osteoclasts by cell-contact-dependent mechanisms, making it a bone-protective type of lymphocytes (98). In physiological conditions, the ratio Th17/Treg is correct and the balance between bone resorption and formation is maintained (99). But in the pathological situations like post menopausal osteoporosis; the balance is disturbed, where there is an increase in Th17 population and decrease in Tregs (100). This change increases osteoclast activity, and decreases osteogenesis, resulting in progressive bone loss (101). An analysis of postmenopausal female patients found increased frequencies of peripheral Th17 cells and IL-17 production and decreased frequencies of Tregs compared with premenopausal women, which may support the role of estrogen deficiency in the promotion of bone loss via increased Th17/Treg ratio (23). More direct animal evidence shows that OVX rats have an increase in Th17 and a decrease in Tregs (102). The treatment with a synbiotic (prebiotic+probiotic) for 12-weeks, however, enhanced the number of SCFA-producing bacteria in the gut, the number of Tregs and reduced the number of Th17s, thus restoring the Th17/Treg balance in the bone marrow and enhancing bone formation. Moreover, supplementation with certain probiotics (e.g., Lactobacillus fermentum, L. rhamnosus) alone was demonstrated to enhance the percentage of Tregs and production of IL-10 in animal model, in which the effect was dependent on SCFA production. These data have highlighted the role of gut microbiota and their metabolites (including butyrate) in regulating Th17/Treg balance, which is important for bone metabolism: beneficial microbes and metabolites promote Treg induction and Th17 suppression, reducing osteoclastogenesis and increasing osteogenesis (103). Dysbiosis or barrier dysfunction, on the other hand, predispose towards a predominant bone resorption, mediated by the Th17. More specifically, an OVX mouse study demonstrated microbiome-dependent trafficking of intestinal TNF-producing T cells and Th17 cells to the bone marrow; blockade of their egress or bone-marrow influx attenuated estrogen-deficiency-induced bone loss (84). This provides mechanistic preclinical evidence and should be distinguished from the associative immune findings reported in postmenopausal women.

Th17 and Treg cells are able to regulate bone cells in several ways at the molecular level. Th17 cells are a major source of RANKL that directly binds to RANK expressed on osteoclast precursors, which results in their differentiation and maturation (104). More significantly, IL-17 produced by Th17 cells interacts with osteoblasts and other target cells to increase RANKL expression and upregulate RANK on osteoclasts and thus amplifies pro-osteoclastogenic signals (105). IL-17 has also been reported to inhibit bone formation by blocking Wnt signaling in osteoblasts, which inhibit osteogenic differentiation and mineralization (106). Moreover, cytokines secreted from Th17 cells (IL-17, IL-22 and IFN-γ) can activate macrophages and osteoblasts to secrete pro-inflammatory cytokines (TNF-α, IL-1β, IL-6), which can also promote osteoclast activity and impair osteoblast function (107). Therefore, the increased activation of Th17 cells is associated with increase in bone resorption and decrease in bone formation, which is closely related to the occurrence and development of osteoporosis (108). By contrast, Treg cells suppress osteoclast activities and maintain bone mass in several ways. The high surface expression of cytotoxic T-lymphocyte-associated antigen 4 (CTLA-4) on Tregs can bind to co-stimulatory molecules on bone marrow stromal cells, reducing their RANKL expression and thereby attenuating osteoclastogenic stimulation (109). Tregs also secrete anti-inflammatory cytokines such as TGF-β, IL-10, and IL-35, which can activate osteogenic MAPK and Smad signaling pathways to promote bone formation and stimulate osteoblasts to secrete the decoy receptor osteoprotegerin (OPG), while suppressing the production of RANKL and M-CSF. Notably, Tregs can directly inhibit osteoclast precursor differentiation via cell-contact-dependent mechanisms: *in vitro* co-culture experiments demonstrate a dose-dependent reduction in osteoclast formation as the ratio of Tregs to osteoclast precursors increases (110). In a mouse model of inflammatory arthritis, adoptive transfer of exogenous Treg cells significantly increased bone mineral density (111), raising trabecular bone volume fraction (BV/TV) from approximately 7.2% to 16.2%, thereby validating the therapeutic potential of Tregs for mitigating bone loss. Moreover, a small clinical trial reported that pharmacological expansion of peripheral Tregs (using low-dose IL-2 (112), increasing Treg frequency from 1.0% at baseline to 2.4–3.0%) was accompanied by an increase in bone formation markers, suggesting that enhancing Treg function may represent a novel osteoanabolic strategy (113).

In summary, the imbalance in the Th17/Treg axis is a pivotal immune mechanism in the pathogenesis of osteoporosis (114), particularly pronounced in the context of estrogen deficiency-driven postmenopausal bone loss, which skews immunity toward a pro-inflammatory state (115). Restoring this balance—for example, through probiotics or cytokine-based therapies that expand Tregs and/or suppress Th17 cells—holds promise for mitigating bone loss and improving bone microarchitecture. Within the framework of the gut-immune-bone axis, the gut microbiota profoundly influences bone metabolism by modulating the Th17/Treg equilibrium: a beneficial microbiome and its metabolites reduce intestinal permeability and pro-inflammatory signaling, thereby limiting Th17-driven osteoclastic activity (116, 117). Conversely, dysbiosis promotes Th17-mediated inflammatory osteoporosis, also referred to as immunoporosis (118) (**Figure 1**).

**Figure 1:**
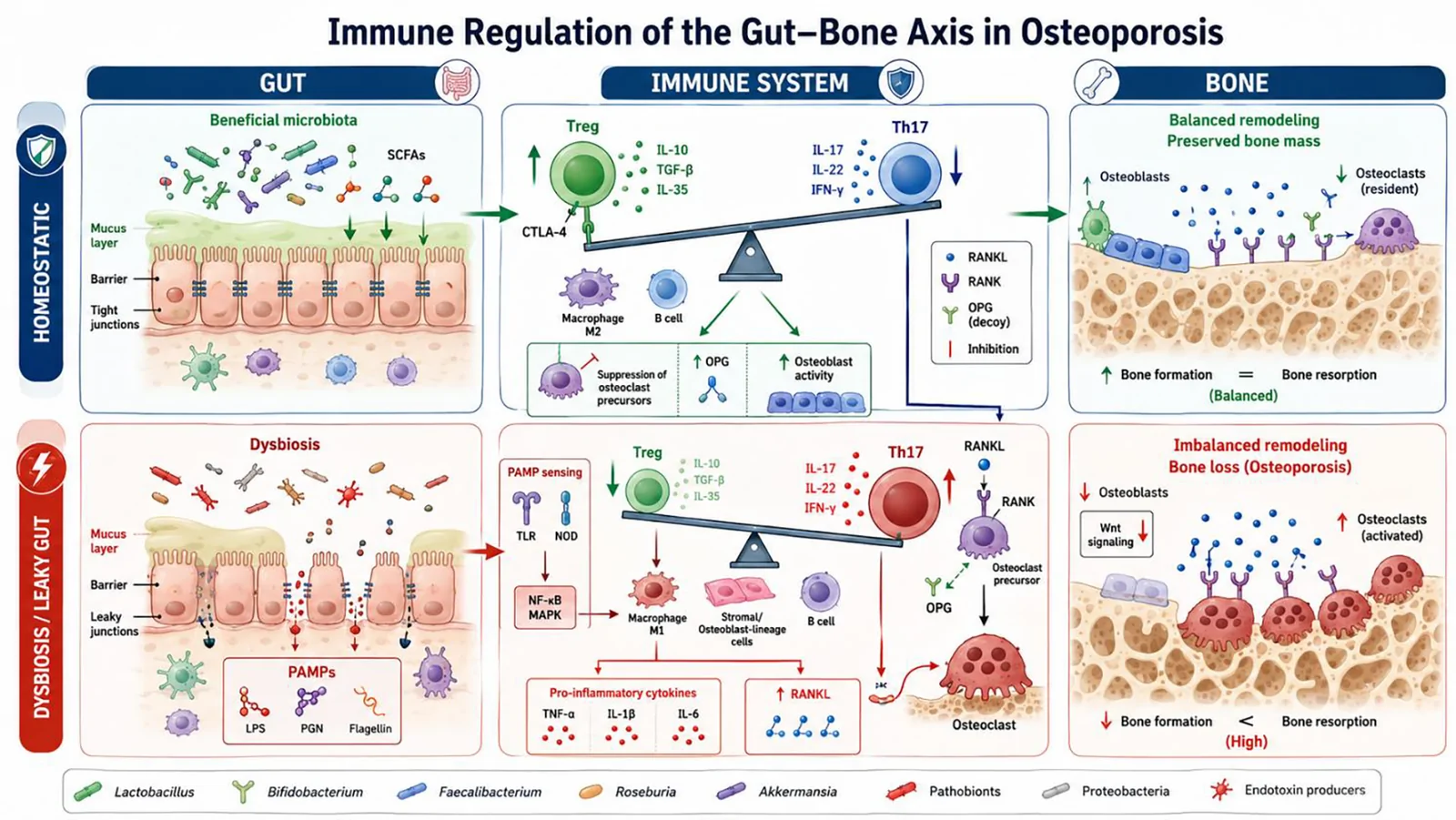
Immune regulation of the gut–bone axis in osteoporosis. Under homeostatic conditions, a beneficial gut microbiota and its metabolites, particularly short-chain fatty acids (SCFAs), support intestinal barrier integrity and promote a Treg-dominant immune environment, thereby suppressing osteoclastogenesis and maintaining balanced bone remodelling. By contrast, dysbiosis and increased gut permeability permit the translocation of pathogen-associated molecular patterns (PAMPs), which activate inflammatory signalling pathways and drive a Th17/Treg imbalance, increased RANKL-mediated osteoclast activity, impaired osteoblast function and, ultimately, bone loss.

### B cells and osteoporosis

B lymphocytes are also involved in the regulation of bone remodeling and the role that they play is dependent on the physiological or pathological environment (119). The young or homeostatic state is a bone-protective state of B cells. Immature B cells express a large amount of osteoprotegerin (OPG), a decoy receptor of RANKL that is a potent inhibitor of osteoclast formation (120). But under conditions of aging or chronic inflammation, the B-cell phenotype changes; they can become fewer in number and they are more likely to produce RANKL rather than OPG, thus changing the equilibrium of remodeling towards bone resorption (121). Under some circumstances, notably when estrogen is deficient, B cell numbers paradoxically increase and become highly activated (122). For example, in the postmenopausal state, bone marrow B cells increase their numbers in response to stromal cells that secrete high levels of the chemokine CXCL12, and B cells also produce significant amounts of the cytokine granulocyte colony-stimulating factor (G-CSF) (123). G-CSF can synergize with RANKL to stimulate the proliferation and differentiation of osteoclast precursors, accelerating osteoclastogenesis (124). Concurrently, pro-inflammatory mediators released by activated B cells inhibit osteogenesis; for example, B cell-derived chemokines such as CCL3 and TNF-α can directly suppress osteoblast differentiation (125). *In vitro* studies demonstrate that LPS-stimulated, activated B cells interfere with osteogenic differentiation of mesenchymal stem cells via the Notch signaling pathway (126), reducing the expression of the master osteogenic transcription factor RUNX2 by more than half. Therefore, in a chronic inflammatory milieu, B cells tend to become pro-osteoclastogenic and anti-osteogenic, potentially explaining the altered B cell phenotypes observed in patients with rheumatic diseases or senile osteoporosis. Conversely, a regulatory B cell subset (Bregs) plays a protective role in bone mass maintenance. Bregs secrete high levels of the anti-inflammatory cytokine IL-10 (127), which suppresses inflammation and osteoclast activity. In a mouse study, stimulation of Breg expansion led to a significant reduction in Th17 cells, an increase in serum IL-10 (128), a decrease in IL-17, and improved trabecular bone structure (reduction in trabecular separation from 0.27 mm to 0.15 mm), demonstrating that Breg-derived IL-10 can inhibit osteoclastogenesis and slow bone loss. This suggests that enhancing Breg function may help counteract inflammatory bone loss. Taken together, B cells exert a dual influence on bone metabolism, capable of either protecting the skeleton or promoting bone loss depending on the prevailing physiological and pathological signals (129). While the direct influence of the gut microbiota on B cell function in the context of bone is less studied, given the microbiome’s profound effect on systemic inflammation and IL-10 production (130), it is plausible that it indirectly shapes B cell-mediated osteoimmune regulation. For instance, some probiotics that trigger the secretion of IL-10 in intestine could be beneficial for the maintenance of bone mass, as they would bring about a Breg phenotype. Therapeutic approaches to osteoporosis could include attempts to “re-educate” B cells to be less pro-osteoclastogenic and more immunomodulatory Bregs to preserve the skeleton. For instance, Sapra et al. showed that IL-10-producing Bregs suppress osteoclastogenesis and mitigate OVX-induced bone loss.

### Macrophages and osteoclastogenesis

The macrophages, which are essential cells of innate immunity, have a complex role in the pathogenesis of osteoporosis. Macrophages have been generally termed as M1 pro-inflammatory or M2 anti-inflammatory, depending on their functional phenotype (131). M1 macrophages secrete a wide range of pro-inflammatory cytokines (TNF-α, IL-1β, IL-6, etc.) which mainly drive the osteoclastogenesis and block bone formation in the skeletal context (132). On the other hand, M2 macrophages release anti-inflammatory and reparative factors (IL-10, TGF-β, BMP-2, etc.), which play an inhibitory role for osteoclast generation and promote osteogenesis (133). In chronic inflammatory states and in estrogen deficiency, polarization favoring the M1 phenotype prevails, and the factors secreted by the polarized M1 macrophages lead to hyperactivation of the osteoclasts, thus contributing to osteoporosis (134).

Specifically, M1 macrophages release abundant TNF-α, which directly acts on osteoclast precursors. TNF-α upregulates RANK receptor expression on precursor cells and synergizes with RANKL signaling to potently enhance osteoclast differentiation via pathways such as NF-κB and PI3K/Akt. TNF-α also targets mesenchymal stem cells, suppressing their osteogenic differentiation while promoting adipogenesis—a mechanism involving inhibition of ERK and JNK signaling and upregulation of the P2Y2 receptor—thereby reducing bone formation (135). IL-1β secreted by M1 macrophages stimulates osteoblasts to produce PGE_2_ and upregulate RANKL expression, indirectly fueling osteoclast activity. IL-6 also promotes osteoclastogenesis and inhibits osteogenesis; its complex with the soluble IL-6 receptor activates the gp130/JAK-STAT3 and NF-κB pathways (136), amplifying osteoclast differentiation even in the presence of low RANKL levels, while simultaneously interfering with Wnt signaling in osteoblasts (137). Additionally, M1 macrophages can activate the NLRP3 inflammasome, leading to the release of IL-1β, which further exacerbates the pro-inflammatory and pro-osteoclastic bone marrow microenvironment (138). It is worth noting that M1 cells also produce factors like IFN-γ and IL-12, which can, under certain conditions, inhibit osteoclastogenesis, partially counterbalancing their pro-resorptive effects (139). Overall, however, the pro-osteoclastogenic signals from M1 macrophages predominate in conditions like osteoporosis (140).

Conversely, M2 macrophages are considered “bone-reparative” due to their anti-inflammatory and pro-regenerative properties (141). M2 macrophages can be further subdivided into subtypes (M2a, M2b, M2c). Classical M2a cells, induced by IL-4/IL-13, secrete factors like BMP-2, IL-10, and TGF-β that promote bone matrix formation and tissue regeneration (142). In an arthritis model, injection of M2a macrophages was shown to reduce synovial inflammation and cartilage destruction while promoting tissue regeneration (143). M2c macrophages (induced by IL-10 or glucocorticoids) are involved in clearing necrotic debris and matrix remodeling during early healing. Overall, M2 cells inhibit osteoclastogenesis and stimulate osteogenesis through the secretion of IL-10, TGF-β, and other factors, and are considered to have a positive impact on bone homeostasis (144). Several nanomaterials and pharmacological agents have been explored for their ability to promote M2 polarization to facilitate bone repair. For example, nano-hydroxyapatite particles can skew macrophages toward an M2 phenotype, increasing IL-10 secretion and thereby promoting osteogenic differentiation of MSCs (145). Exendin-4, a GLP-1 receptor agonist, can induce M2 polarization by the cAMP/PKA/STAT3 pathway to increase the level of TGF-β1 and recruit MSCs to the bone surface for bone formation (146).

An imbalance of M1/M2 ratio is an important pathological mechanism in osteoporosis (147). In postmenopausal osteoporosis, for example, estrogen deficiency induces M1 polarization and thus an increase in the production of TNF-α and other factors, creating a vicious ‘inflammation-bone loss’ cycle (148). M1 derived signals directly stimulate bone resorption and also inhibit M2 phenotype to prevent the development of anti-inflammatory forces, thus continuing a vicious cycle of increasing inflammation and bone loss (149). This imbalance was recently documented in single-cell RNA sequencing experiments, which showed increased numbers of M1-like clusters and relative decrease in M2-like clusters in the bone marrow macrophage lineage in osteoporotic models (150). Hence, specific intervention to inhibit activity of M1 or augment M2 polarization is a promising approach. For instance, blockade of the main effector molecule of the immune system, TNF-α (with biologics), in patients with rheumatoid arthritis reduces the inflammation in the joints as well as enhancing BMD (151). Likewise, inhibitors of IL-1 or IL-6 have proven to be effective in reducing bone loss in inflammatory disease patients (152). The above observations validate the idea that modulation of pro/anti-inflammatory signaling of macrophages can impact on systemic bone metabolism (153). Importantly, the gut microbiota is also intertwined in this process as gut-derived LPS can promote M1 polarization through TLR4 signaling, while microbial metabolites such as SCFAs can promote an anti-inflammatory or M2-like polarization (154). Thus, modulation of gut microbiota and improvement of intestinal barrier function, could be a way to reduce circulating pro-inflammatory PAMPs, reduce M1 activity and consequently reduce progression of osteoporosis (155).

To conclude, the immune system is involved in the gut-bone axis in a variety of ways via cells and factors. Together, the Th17/Treg axis of T cells, the RANKL/OPG secretory balance of B cells and the M1/M2 balance of macrophages contribute to the immune microenvironment of bone remodeling (156). The gut microbiota and its metabolites, in turn, have a profound influence on these immunological effectors: the metabolite butyrate favours Tregs and perhaps M2 phenotypes, whereas LPS leads to Th17 and M1 polarization (157). Thus, it is important to understand the cause of osteoporosis and to understand the role of systemic inflammation and gut health on bone metabolism and consider the skeleton as an immune-modulated organ (158). This insight also provides new therapeutic approaches to osteoporosis such as anti-inflammatory or immunomodulatory drugs as a protective approach to bone and probiotics as a means of rebalancing immune cell populations (159). The next section will discuss the influence these factors, along with the results of advanced quantitative MRI, have on bone tissue structure (160) (**Figure 2**).

**Figure 2:**
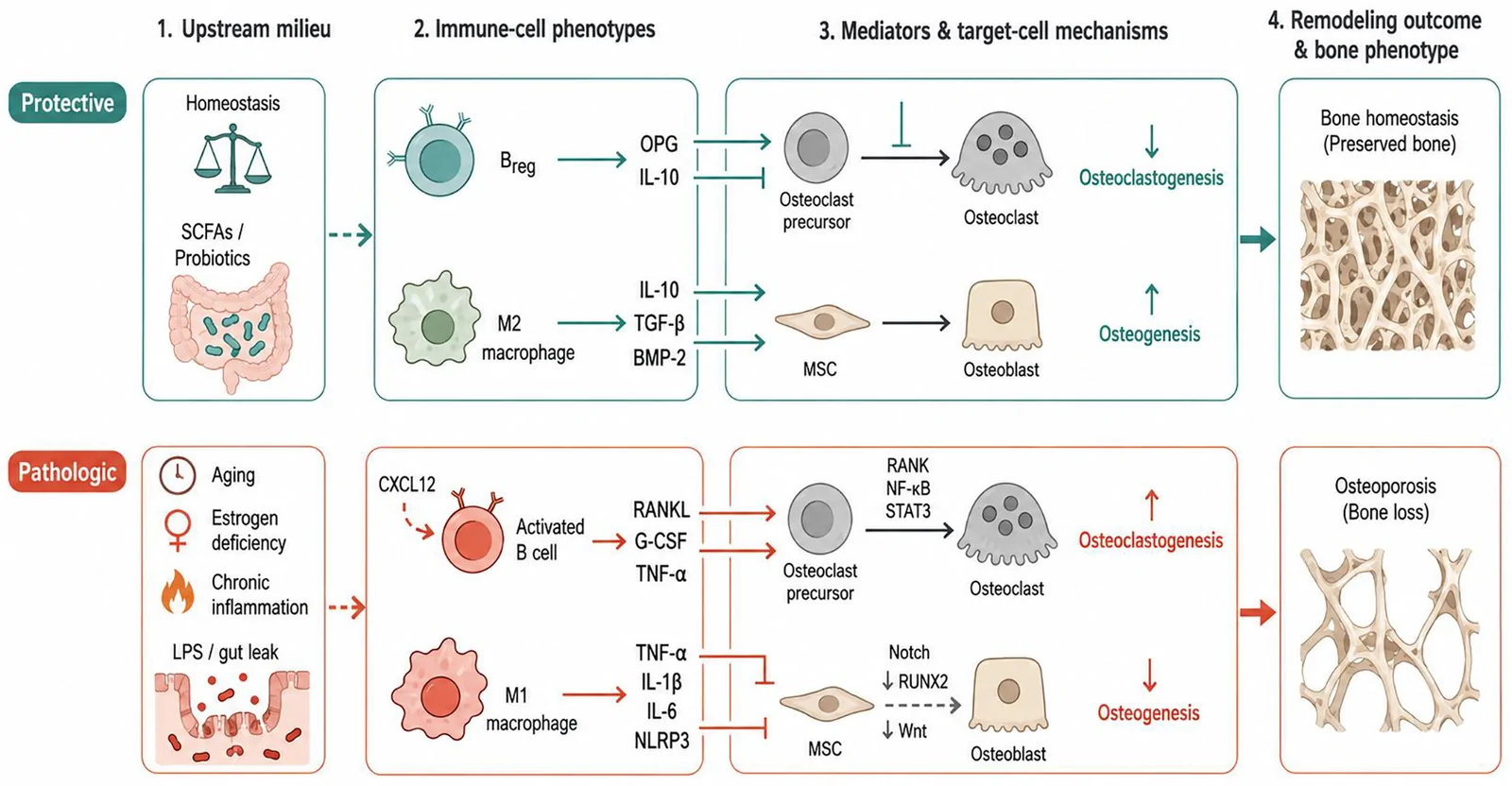
B cell- and macrophage-mediated osteoimmune regulation in osteoporosis. Under homeostatic conditions, B cells and M2-like macrophages protect bone by producing OPG, IL-10, TGF-β, and BMP-2, thereby inhibiting osteoclastogenesis and promoting osteogenesis. In contrast, aging, estrogen deficiency, chronic inflammation, and gut-derived LPS drive activated B cells and M1 macrophages to release RANKL, G-CSF, TNF-α, IL-1β, IL-6, and NLRP3-related inflammatory signals. These mediators enhance osteoclast differentiation while suppressing MSC-to-osteoblast differentiation through RANK/NF-κB/STAT3, Notch/RUNX2, and Wnt-related pathways, ultimately shifting bone remodeling toward bone loss and osteoporosis.

## Quantitative MRI assessment techniques and findings in osteoporosis

The use of DXA for bone densitometry is the current clinical gold standard for diagnosis of osteoporosis and fracture risk assessment, but it only gives an areal measurement of the quantity of bone and not a direct measurement of bone microarchitecture or quality (161). The underlying feature of osteoporosis is loss of trabecular network and thinning and porosity of the cortex (162). Thus, imaging methods that would be able to describe the microarchitecture of bone and tissue composition are important for a thorough assessment of bone fragility (163). In recent years, magnetic resonance imaging (MRI), being non-ionizing and able to image both soft and hard tissues, has become a distinctly beneficial tool for osteoporosis research (164). MRI is currently the only completely non-invasive and radiation-free imaging method that can simultaneously assess both trabecular and cortical bone compartments (165). With advances in high-field MRI instrumentation and rapid imaging sequences, the spatial resolution and quantitative capabilities of MRI for skeletal assessment have continually improved, providing novel methods for detecting early osteoporotic changes (166). However, DXA is a low-dose procedure widely used in clinical practice. Its advantages include low cost, short scan time, and well-established reference data for fracture risk. In microbiome studies, quantitative MRI markers like vertebral PDFF or T2* may act as surrogates of marrow inflammation or fat accumulation resulting from gut-induced immune changes. Future trials could use MRI endpoints to evaluate the effects of microbiome-targeted therapies. Within the gut–immune–bone framework, qMRI should be regarded as a downstream skeletal phenotyping platform rather than a direct biomarker of microbiome composition or immune activation. Candidate measures such as vertebral proton-density fat fraction (PDFF), marrow T2/T2* metrics, trabecular BVF/Tb.Th/Tb.N/Tb.Sp, and UTE-derived cortical indices may capture structural or marrow-compositional consequences that accompany microbiome- or inflammation-related changes. Their specificity for the gut–immune–bone axis, however, has not yet been established and requires prospective multimodal validation.

### MRI assessment of bone microarchitecture

High-resolution MRI can directly image the marrow phase within the trabecular lattice (the spaces between trabeculae, which contain marrow consisting of fat and water and appear hyperintense on conventional MRI) (167). This allows for the indirect inference of trabecular morphology (168). Through appropriate sequence selection and post-processing, a suite of parameters corresponding to bone microarchitecture can be quantified, including bone volume fraction (BVF), trabecular thickness (Tb.Th), trabecular separation (Tb.Sp), and trabecular number (Tb.N) (169). These parameters correlate well with measurements from gold-standard high-resolution peripheral quantitative computed tomography (HR-pQCT) and have demonstrated value in distinguishing osteoporotic patients from healthy individuals (170). For example, in a study of proximal femoral bone microarchitecture using 3T MRI, MRI-derived bone volume fraction (BVF), trabecular thickness (Tb.Th), and trabecular number (Tb.N) were significantly different between healthy individuals and those with osteoporosis, offering a more sensitive reflection of trabecular deterioration compared to DXA-derived BMD (171). Importantly, quantitative MRI-derived microstructural parameters provide superior fracture risk prediction compared to BMD alone (172). Early studies found that adding MRI-based trabecular structural analysis significantly enhanced the predictive power for fractures (173). In a study focusing on the hip, the area under the curve (AUC) for predicting femoral neck fracture using high-field MRI-derived trabecular parameters was substantially higher than that for DXA-BMD (174). Thus, MRI serves as a valuable adjunct that can address the limitation of DXA, which may fail to identify individuals at high risk for skeletal fragility (175).

Various MRI methods are employed to assess bone microarchitecture, with spin-echo and gradient-echo sequences being the earliest applied for imaging the distal radius and calcaneus (176). These uses have been expanded to include deeper skeletal locations, like the tibia and spine, thanks to technological improvements (177). Optimized gradient-echo imaging sequences on current 3T clinical MR imaging platforms can achieve spatial resolution of around 0.2–0.3 mm which is typically sufficient for the resolution of trabecular structures (178). Further decreasing the dimensions of the pixel with ultra-high-field 7T MRI allows for the visualization of finer trabecular network (179). A 7T MRI study for example, was able to successfully report a gradient in trabecular architecture from epiphysis to metaphysis of the distal radius and find significantly higher BVF and Tb. The N content of the distal part was lower than that of more proximal parts (180). These technological developments are helping to make MRI more and more of a mature quantitative imaging technique for bone microarchitecture. Nevertheless, the spatial resolution of MRI remains limited, and adjacent trabeculae cannot always be fully resolved (181). Consequently, derived quantitative parameters often rely on model-based assumptions and calculations, and a universally accepted standard is still lacking (182). Despite this, numerous studies have validated the utility of MRI-quantified trabecular parameters in reflecting osteoporotic pathology and monitoring therapeutic response (183). For example, MRI of the distal tibia in patients with chronic kidney disease reveals significant trabecular deterioration that correlates with disease stage (184). Similarly, in a model of glucocorticoid-induced osteoporosis, MRI detected decreases in trabecular number and thickness alongside an increase in marrow adiposity, consistent with the state of elevated bone resorption (185). In summary, quantitative MRI microstructural analysis provides invaluable, non-invasive information for the early detection of osteoporosis (186).

Notably, MRI can quantify not only trabecular but also cortical bone status. Conventional MRI sequences, due to rapid signal decay from short T2 components, render cortical bone virtually signal-void (black), making assessment of cortical thickness and porosity challenging (187). However, the recent development of ultrashort echo time (UTE) imaging techniques has significantly improved this capability (188). UTE sequences, by reducing echo times to the microsecond scale, can capture signal from cortical bone before it decays, thereby “visualizing” the cortex on MRI (189). Based on UTE imaging, quantitative and qualitative indices of cortical bone can be extracted, including cortical bone thickness, porosity, and degree of mineralization (190). For instance, UTE-derived quantitative susceptibility mapping (UTE-QSM) has been used to quantify cortical bone mineral density, showing good agreement with quantitative computed tomography (QCT) results (191). Studies indicate that UTE-QSM can reliably assess volumetric BMD (vBMD) in both cortical and trabecular compartments, holding promise for the quantitative diagnosis of osteoporosis (192). Furthermore, UTE combined with dual-echo techniques can estimate local bone volume fraction (BV/TV), showing strong correlation with high-resolution micro-CT findings (193). Thus, UTE-MRI opens a new window for assessing cortical bone quality, enabling a more comprehensive MRI-based evaluation of the skeleton (194). To sum up, quantitative MRI can provide information on trabecular and cortical bone, both in a single measurement, without the use of ionizing radiation (high resolution trabecular imaging and UTE for cortical bone assessment). This benefit of not needing to be invasive is especially important for patients who need to be monitored over a period of time (195).

### MRI analysis of bone marrow composition and metabolism

In addition to structure, metabolic state of the skeleton is an important aspect of osteoporosis (196). For example, bone marrow adiposity and bone loss have a strong association (197). Because MRI is sensitive to fat signals and water signals, it is possible to quantify the composition of bone marrow and get metabolic information which cannot be obtained by DXA (198). MRS and water-fat separation imaging using a chemical-shift technique (e.g., Dixon techniques) are common methods to measure the bone marrow fat fraction (BMFF) (199). Bone marrow adiposity is found to be significantly increased in patients with osteoporosis (or “individuals with osteoporosis”) and is inversely associated with bone mass in studies (200). An MRI study of the distal radius, for instance, revealed that postmenopausal osteoporotic women had significantly higher BMFF and lower T2 relaxation time than healthy controls, suggesting that there was greater infiltration of marrow fat and less water content (201). Additional studies demonstrate that higher BMFF levels are frequently associated with BMD loss prior to its detection, suggesting that it may be a valuable early risk marker for osteoporosis (202). Thus, detection of marrow adiposity by MRI may help to identify high-risk individuals prior to the presence of significant bone loss by DXA (203). MRI fat-water separation techniques (e.g., IDEAL or Dixon) can generate fat fraction maps in a single acquisition, allowing for the assessment of fatty infiltration across multiple skeletal sites (204). Clinical studies have established strong correlations between marrow fat content in the lumbar spine and proximal femur and patient age, BMD, and the risk of fragility fracture (205). Additionally, MRS can differentiate the degree of fatty acid unsaturation and detect specific metabolite peaks, offering further insights into bone marrow lipid metabolism (206). Collectively, MRI-based marrow analysis provides direct *in vivo* evidence for the pathological shift in the differentiation of mesenchymal stem cells from the osteoblastic to the adipocytic lineage that characterizes osteoporosis (207). In osteoporotic conditions, this relationship between osteogenesis and adipogenesis is altered, with increased marrow adipocytes (203). MRI can measure this process allowing it to be used to monitor treatment responses (208).

Interestingly, chemical shift-encoded MRI also has a role in distinguishing between the benign osteoporotic fractures and malignant bone metastases (209). Both can cause abnormal signals in the marrow on MRI but CSE-MRI quantification shows a smaller decrease in local marrow fat fraction in osteoporotic compression fracture than in metastatic lesions where tumor cells are usually found to replace the fatty marrow (210). Thus, the use of BMFF threshold analysis is useful to improve the diagnostic accuracy in the discrimination of these entities (211). Clinical studies have demonstrated that quantitative evaluation of the marrow fat fraction by MRI enables the distinction of osteoporotic from pathological fractures and thereby prevents the misdiagnosis and wrong treatment (212). This highlights the clinical usefulness of quantitative MRI in orthopedics (213).

### Emerging frontiers and clinical significance of quantitative MRI

The use of MRI in osteoporosis is increasing in depth and breadth as image processing and analytical tools are continually developing (214). For example, MRI statistical parametric mapping (SPM) can be performed to voxel-wise compare maps of microstructural or fat distribution throughout the bone, and detect region-specific microscopic osteoporotic changes (215). Several investigations have used MRI-derived trabecular structural maps to estimate the mechanical competence of bone and to predict fracture risk, through finite element analysis (216). In addition, AI and radiomics are increasingly adopted to analyze MR images of osteoporosis (217). By using high throughput feature extraction from the MRI data, predictive models can be developed with a greater accuracy to separate the osteoporotic patients from healthy ones and to predict the occurrence of fractures (218). MRI-based radiomics models have been reported to be able to characterize bone geometry, material distribution, and microstructural features quantitatively, which can help in diagnosis and therapy in osteoporosis (219). These cutting-edge techniques have increased MRI sensitivity and specificity, and are leading the shift in the management of osteoporosis from a purely radiographic diagnosis to digital risk assessment (220).

Finally, quantitative MRI offers a new understanding and a valuable toolbox for the assessment of osteoporosis (221). It is able not only to identify structural changes in the bones (modifications of trabecular and cortical bone) but also to identify metabolic changes within the bone (e.g. increased marrow adiposity), which is a good supplement to the limitations of the traditional DXA assessment (222). MRI combines assessment of bone microarchitecture and marrow composition which can help detect a decrease in bone quality before bone fractures occur, allowing timely intervention to prevent fragility fractures (223). Furthermore, MRI could be used as an efficacy endpoint in pharmaceutical trials to assess the effect of anti-resorptive and/or osteoanabolic drugs on the trabecular structure and marrow fat and thus provide multidimensional evidence on the efficacy of the drug (224). As imaging data from different centers become more standardized and the speed and spatial resolution of MRI continue to improve, it is expected that quantitative MRI will become an important additional tool for the clinical assessment of osteoporosis for the purposes of personalized patient care and fracture risk prediction (225).

As is described in **Figure 1**, gut microbiota, immune system and bone metabolism are linked together through an intricate network, which is a functional integration of the multiple systems, known as the ‘gut-immune-bone axis’ (12, 226). During health, gut-dependent metabolites contribute to a healthy barrier function and immune balance, and immune cells regulate bone remodeling in the right way. Immune imbalance in pathological states (e.g. dysbiosis or chronic inflammation) can trigger too much osteoclast function and less bone formation; in addition, the gut itself may also become a source of systemic inflammation; these three can act together to promote osteoporosis (89, 226). Given this systems perspective, recent advances in research include a systems-level understanding of and intervention in osteoporosis, which goes beyond a bone-centric perspective to include the gut ecosystem and immune network. The following are important developments and future trends in this area:

Multi-Omics Integration and Network Analysis: In order to dissect the gut-immune-bone axis in a comprehensive manner, researchers are utilizing multi-omics technologies such as metagenomics, metabolomics, metatranscriptomics and single cell sequencing to integrate them with the various biological systems (227). For instance, it is now possible to profile the gut microbial makeup, the serum metabolome, and the bone marrow immune cell transcriptome at the same time to build cross-system correlation networks and identify key microbial-metabolite-immune nodes (228). A study in aged osteoporotic mice has combined the -omics approach of 16S rRNA sequencing with targeted metabolomics to reveal specific microbial taxa (such as Parabacteroides) and metabolites (HDCA bile acid) that strongly correlate with bone mass, and demonstrated the causal role of aged microbiota in bone loss by fecal microbiota transplantation experiments (229). A global perspective is very important for the understanding of multiple convergent pathways (nutritional, immune or endocrine) by which the gut microbiota can influence bone metabolism (230). The next steps should include large human cohort studies and the use of multi-omics and bioinformatics modelling to create a ‘cross-system regulatory network map of osteoporosis’, that could be used to identify practical intervention points and new biomarkers (231).

A major challenge is the identification of keystone strains and metabolites in the diverse gut microbial ecosystem that have a large effect on bone metabolism. An example of such research is the age-related decrease in the beneficial bacterium P. goldsteinii, and the decrease in HDCA, a bone-protective metabolite produced by P. goldsteinii, that can be prevented by supplementation (232). In a similar way, defined microorganisms able to produce butyrate and indole play a key role for the Treg/Th17 balance and bone mass preservation (233). One way is to explore the many datasets of microbiomes for the presence of strong correlators of skeletal phenotypes as probiotics or pathobionts. Animal models are necessary to confirm the causal role of these candidate microbes on bone mass and metabolomics will help to elucidate mechanisms of action of microbial products such as SCFAs, IAA and secondary bile acids (234). Identification and validation of a keystone microbe/metabolite could lead to the development of a biological therapeutic or functional food for human intervention (235). For example, several probiotic strains (such as Bifidobacterium and Lactobacillus) have been shown to increase calcium absorption in the host and decrease inflammation and are considered to be potential adjunctive therapy for osteoporosis (236). Oral probiotics, dietary modulation, or supplementation with specific metabolites (e.g., prebiotics to increase SCFA production) may be clinically relevant approaches to targeting these key nodes (237).

Microbiome-based therapeutics: Microbiome-directed interventions remain of considerable translational interest, but current clinical evidence is heterogeneous. Probiotic effects appear to be strain specific and may depend on viable dose, treatment duration, baseline microbiome configuration, diet, adherence, host metabolic and immune phenotype, and the ability of administered organisms to persist or exert sustained functional effects. Human trials illustrate this variability: a 6-month trial of L. fermentum SRK414 reported maintenance of osteocalcin and an increase in femoral-neck BMD (24), whereas a 2-year randomized trial of L. reuteri 6475 did not reduce bone loss or bone-turnover markers (25), and a 48-week trial of L. brevis HA-112 plus L. paracasei HA-274 did not significantly improve femoral-neck BMD or fracture-risk estimates (26). These findings suggest that responder/non-responder heterogeneity, strain selection, dosing, treatment duration, and functional engraftment must be addressed before probiotics can be considered established osteoporosis therapies. Manufacturing consistency, viability, quality control, and regulatory classification also remain important translational challenges. Accordingly, probiotics are best regarded at present as investigational or adjunctive strategies rather than replacements for established osteoporosis treatment. More advanced approaches include engineered probiotics designed to deliver defined metabolites or functions, although feasibility has so far been demonstrated mainly in preclinical models (238, 239). Fecal microbiota transplantation (FMT) is also an important experimental approach for testing microbiome causality. Preclinical studies indicate that transplantation of microbiota can transfer skeletal phenotypes between donors and recipients, including acceleration of bone loss by osteoporotic donor microbiota and protection by younger or metabolically healthier donor communities. However, FMT for osteoporosis remains highly experimental. Major concerns include donor selection and screening, transmission of infectious agents or undesirable microbial traits, donor-to-donor variability, uncertainty regarding the durability and reproducibility of engraftment, and the absence of standardized osteoporosis-specific protocols. Current regulatory frameworks largely restrict routine FMT use to defined gastrointestinal indications; therefore, clinical translation to skeletal disease will require controlled trials with long-term safety surveillance and standardized microbiome, immune, and skeletal endpoints.

Immune Targeting and Anti-inflammatory Interventions: As the immune system is clearly central to osteoporosis, there is great potential for therapies targeting the immune system (240). However, there is evidence that current anti-inflammatory biologics are beneficial for bone in inflammatory osteoporosis (241). Biologics that target TNF-α, IL-6R or IL-17A have been shown to not only curb the inflammatory disease itself, but also halt secondary bone loss and improve BMD in patients with diseases such as RA and AS (242). These results suggest that, in appropriate clinical scenarios, such cytokine inhibitors may benefit osteoporosis patients (1). For example, in postmenopausal women at high fracture risk who exhibit a chronic inflammatory state (e.g., from a co-existing rheumatic disease), drugs blocking the IL-17 pathway could theoretically mitigate excessive osteoclast stimulation (243). A study in OVX mice confirmed that genetic knockout or neutralization of IL-17 partially prevented estrogen-deficiency-induced bone loss (244). Furthermore, clinical trials in patients with psoriasis have shown that anti-IL-17A antibody therapy over 6–12 months can stabilize or slightly increase BMD (245). On the other hand, enhancing bone-protective immune responses is another important avenue. For instance, expanding Tregs with low-dose recombinant IL-2 or stimulating IL-10 production with a specific vaccine adjuvant (FPD) have both been shown to improve bone mass in animal models (246). A recent report showed that in a model of myeloma bone disease, weekly administration of low-dose IL-2 remodeled the bone marrow immune microenvironment, increasing the number of CXCR3^+^ Tregs and IL-10 levels, and as a result, reversed bone loss in mice (247). This validates the feasibility of “pro-regulatory immunotherapy.” Future directions may include more targeted immune interventions, such as blocking chemokine pathways to prevent osteoclast precursor recruitment, using checkpoint agonists to enhance Treg function in bone marrow, or employing CAR-T cell technology to deplete pathologically activated bone-destroying cells. These immunotherapies are promising but need a balance between the systemic immune response and the effects on bone and are expected to be developed in the future through interdisciplinary cooperation (248).

Moving Advanced MRI and Other Advanced Technologies to the Clinic: The advanced imaging techniques outlined above, including qMRI, are increasingly relevant to translational osteoporosis research (249). Radiomics and artificial intelligence may integrate MRI-derived microstructural and marrow-composition information with clinical risk factors and other modalities such as HR-pQCT (250–252). In the context of the gut–immune–bone axis, the most appropriate role for qMRI is as a longitudinal downstream endpoint rather than a stand-alone microbiome biomarker. Future microbiome-targeted or immunomodulatory trials could pair changes in vertebral or femoral PDFF, marrow T2/T2*, trabecular BVF/Tb.Th/Tb.N/Tb.Sp, and UTE-derived cortical measures with microbial composition, circulating metabolites, inflammatory cytokines, and bone-turnover markers such as CTX and P1NP. Such multimodal designs could test whether biological changes in the microbiome–immune compartment precede or parallel measurable changes in marrow composition and bone microarchitecture. Importantly, these imaging endpoints remain candidate surrogate markers and require prospective validation for responsiveness, reproducibility, and clinical relevance before they can be used as treatment-monitoring biomarkers specific to microbiome-directed therapy.

In summary, the study of the “gut-immune-bone axis” is making consistent progress from discovery to clinical translation. Understanding of the pathogenesis of osteoporosis has grown from being a disease of bone metabolism to a systemic imbalance of networks (253). This view enables identification of new osteoporosis therapeutic targets and offers novel strategies to treat recalcitrant osteoporosis (254). In the coming years, use of probiotic formulations, bone-protective microbiota transplants and immune cell modulators in combination with traditional anti-osteoporotic drugs with multiple targets may become reality, thus creating a multi-targeted “microbiota-immune-bone” therapeutic paradigm. At the same time, cutting-edge technologies such as MRI will make it possible to evaluate skeletal health and the effectiveness of treatment sooner and more accurately which will help advance personalized care. As research deepens, a more comprehensive picture of the integrated physiology and pathology encompassing the gut microbiome, immune network, and bone metabolism will emerge, bringing new hope for conquering osteoporosis (255).

## Current challenges and limitations

Despite the rapidly growing body of evidence supporting the gut–immune–bone axis, several limitations should be considered when interpreting the current literature. First, much of the mechanistic evidence remains preclinical, including germ-free models, antibiotic-treated animals, ovariectomized rodents, FMT experiments, and *in vitro* bone-cell systems. These models are valuable for establishing biological plausibility and potential causality, but their findings cannot be assumed to translate directly to human osteoporosis. Human intervention trials remain relatively few, often have modest sample sizes, and show substantial heterogeneity in participant characteristics, probiotic strains, dose, treatment duration, adherence, baseline microbiome composition, and skeletal endpoints. Recent randomized trials have reported inconsistent effects on BMD and bone-turnover markers (24–26), emphasizing the gap between promising experimental findings and reproducible clinical efficacy. Second, microbiome studies differ in sample collection and storage, DNA extraction, 16S rRNA versus shotgun metagenomic sequencing, taxonomic resolution, bioinformatic pipelines, and statistical treatment of compositional data. These methodological differences complicate cross-study comparison. Inter-individual microbial variability is also substantial and may be confounded by diet, medications (including antibiotics, proton-pump inhibitors, metformin, glucocorticoids, and anti-osteoporosis agents), age, sex, menopausal status, geography, ethnicity, lifestyle, renal function, diabetes, and inflammatory comorbidities. Third, metabolomic studies are limited by differences in targeted versus untargeted platforms, sample matrices, normalization procedures, and the absence of standardized concentration thresholds for many microbial metabolites. Fourth, osteoporosis itself is heterogeneous: postmenopausal, age-related, glucocorticoid-induced, diabetic, CKD-associated, inflammatory, and cancer therapy-associated bone loss may involve distinct combinations of endocrine, microbial, immune, and marrow mechanisms. Finally, qMRI biomarkers require harmonized acquisition and post-processing across scanners and centers, and their relationship to microbiome-induced immune changes remains largely inferential. Future research should therefore prioritize adequately powered longitudinal human cohorts, randomized intervention trials, sex- and etiology-stratified analyses, standardized microbiome and metabolomic workflows, and integrated measurement of microbial, immune, biochemical, and imaging endpoints. Until such evidence is available, microbiome-directed therapies should be considered investigational or adjunctive rather than established treatments for osteoporosis.
